# Patient-Derived Models of Liver Cancer to Inform Clinical Treatment Paradigms: Recent Updates

**DOI:** 10.1055/a-2779-4984

**Published:** 2026-02-24

**Authors:** Kelley Weinfurtner, Rudra Amin, Nicolas Skuli, Terence P. Gade, David E. Kaplan

**Affiliations:** 1 Division of Gastroenterology and Hepatology, University of Pennsylvania, Philadelphia, Pennsylvania, United States; 2 Department of the Radiologic Sciences, Penn Image-Guided Interventions Lab, Department of Radiology, University of Pennsylvania, Philadelphia, Pennsylvania, United States; 3 Drexel University College of Medicine, Drexel University, Philadelphia, Pennsylvania, United States; 4 Department of Cancer Biology, University of Pennsylvania, Philadelphia, Pennsylvania, United States; 5 Division of Interventional Radiology, Corporal Michael J Crescenz VA Medical Center, Philadelphia, Pennsylvania, United States; 6 Division of Gastroenterology, Corporal Michael J Crescenz VA Medical Center, Philadelphia, Pennsylvania, United States

**Keywords:** hepatocellular carcinoma, cholangiocarcinoma, patient-derived models, organoids, patient-derived xenograft

## Abstract

Primary liver cancer remains a global health challenge due to rising incidence, limited curative options, and poor overall survival. Poor outcomes stem from tumor heterogeneity, limited efficacy of current therapies, and comorbid chronic liver disease. Despite recent advances in immunotherapy and combination treatments, response rates remain low, and predictive biomarkers are lacking. As a result, there is an urgent need for preclinical models that capture the molecular, cellular, and immune landscape of primary liver cancer. This review discusses the strengths and limitations of patient-derived models of liver cancer, including two-dimensional patient-derived cell lines (PDCL), three-dimensional (3D) patient-derived tumor organoids (PDTOs), and patient-derived xenografts (PDXs). While PDCLs and PDTOs enable high throughput studies, they lack a representative tumor microenvironment. PDXs, including PDXs in animals with humanized immune systems, may more effectively mimic tumor–environment interactions but are costly, complex, and still contain mouse stromal cells. Ex vivo tissue culture preserves tissue structure and cell–cell interactions in an immunocompetent environment; however, short duration of viable culture limits broader application. Continued innovation in the development of multicellular three-dimensional culture systems and in vivo humanization strategies will play a critical role in enabling the development of more personalized and effective therapies for primary liver cancer.

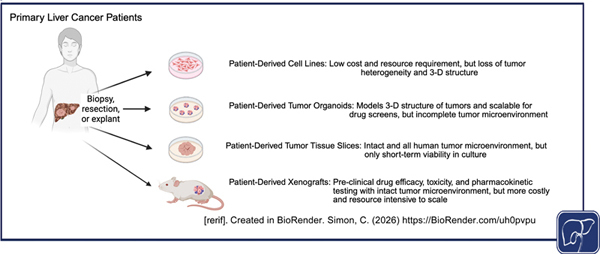

## Introduction

Primary liver cancer is the third leading cause of cancer-related mortality worldwide with both incidence and associated mortality expected to increase by over 50% over the next 20 years. 1 2 Hepatocellular carcinoma (HCC) accounts for approximately 80% of primary liver cancer cases in adults, whereas cholangiocarcinoma (CCA) and combined hepatocholangiocarcinoma (cHCC–CCA) comprise most of the remaining cases. Morbidity and mortality rates remain high for these cancers with overall expected survival less than 2 years. 1 3 This poor prognosis stems from the late stage at presentation, the modest efficacy of locoregional and systemic treatment options, and the chronic liver disease that is heavily comorbid in these patients.

Significant heterogeneity among HCC patients has been a major hurdle in the development of effective treatments. 4 5 6 7 HCC develops in the context of chronic liver disease with over 80% of HCC patients having underlying cirrhosis but from multiple different etiologies that each result in differential impacts on the tumor microenvironment and distinct genetic, epigenetic, and environmental alterations that contribute to the multistep process of oncogenesis. 8 This is evidenced by the mutational landscape of HCCs that demonstrate an average of 30 to 40 mutations per tumor. 9 10 11 Notably, no mutation is observed in more than half of patients except mutations in the TERT promoter, and only 15 to 25% of tumors harbor targetable driver mutations. 7 9 10 11

Over the past decade, considerable progress has been made in developing a growing repertoire of therapeutic options for patients with incurable/unresectable HCC (uHCC) that include both locoregional and systemic therapies. 12 13 Locoregional therapies (LRTs) continue to be the most commonly used treatments for patients with uHCC; however, choice of modality is mostly driven by provider/center experience and resources, as there are no multicenter, randomized controlled trials comparing these approaches nor treatment-specific biomarkers used in clinical practice. 14 15 16 17 Duration of response remains highly variable, and repeated treatments significantly impact liver function over time. Excitingly, there has been a significant expansion of systemic therapy options driven largely by success using immune checkpoint inhibitors (ICIs) in combination therapies. 18 19 20 21 22 23 24 However, clinical trials in this space have highlighted a tremendous unmet need. While some patients exhibit dramatic and durable responses, overall objective response rates remain ≤30%. 22 23 24 Biomarkers of response to ICIs that have been useful in other malignancies have not proven predictive of response in HCC. 25 In addition, each therapeutic combination requires optimization of the scheduling and dosing, as well as appropriate patient selection that takes into account the diversity of molecular alterations and underlying liver dysfunction. Addressing these challenges through human clinical trials alone is infeasible given the vast numbers of potential therapeutic combinations and the prohibitive costs required. Consequently, there is an urgent need for sophisticated translational models that recapitulate the complexity of tumor-intrinsic factors, the tumor immune microenvironment, and underlying chronic liver disease seen in HCC patients.

## Patient-Derived Models of Liver Cancer

**Fig. 1:**
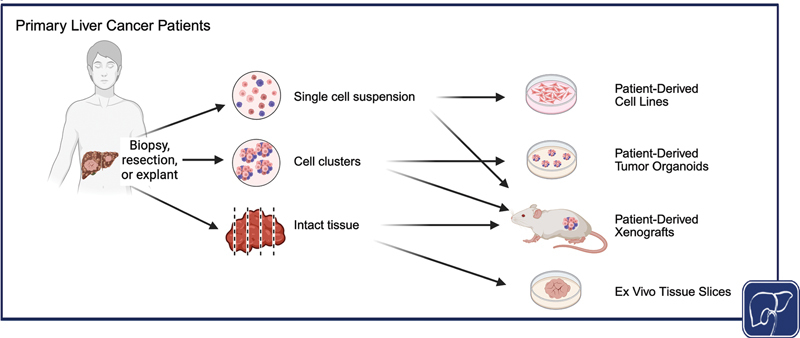
Preclinical models of liver cancer using patient tissue. Schematic representation of different patient-derived model systems. [rerif]. Source: *Created in BioRender. Simon, C. (2026)* *https://BioRender.com/uh0pvpu* .

Historically, two-dimensional cancer cell lines have been the backbone of liver cancer research, as they are relatively low-cost, can be grown *in vitro* as well as *in vivo* in immunocompromised mice, and allow for high-throughput drug screening approaches. Notably, there are several important drawbacks to these models, including the incomplete recapitulation of genetic variants observed in patients, absence of intratumoral heterogeneity, genotypic and phenotypic drift in culture, and lack of tumor microenvironment *in vitro* ( Table 1 ). 26 27 28 29 30 Models designed to address some of these challenges, including novel patient-derived cell lines (PDCL) with coculture systems, patient-derived tumor organoids (PDTO), and patient-derived xenografts (PDX), will be herein reviewed ( Fig. 1 ).

**Table 1:** Comparison of advantages and limitations of patient-derived models

	Patient-derived cell lines	Patient-derived tumor organoids	Patient-derived xenografts
Advantages	(1) Low cost, straightforward protocols (2) Robust system for investigating molecular mechanisms. (3) Easy to scale for high throughput screens	(1) Model 3D structure of tumors including nutrient and oxygen gradients (2) Can culture directly from patient biopsies (3) Scalable for high-throughput screens (4) Maintain molecular and histologic features for at least 1 y in culture	(1) Preclinical drug testing for efficacy, toxicity, and pharmacokinetics (2) Large amount of tissue for experiments (3) Maintain molecular and histologic features through at least five passages (4) Allow for studies of locoregional therapies
Limitations	(1) Loss of intratumor heterogeneity (2) Loss of 3D structure, cell–cell and cell–ECM interactions (3) Lack of tumor microenvironment (4) Higher potential for genotypic/phenotypic drift (5) Low take rates (6) Poor representation of well differentiated tumors	(1) Require more technical expertise and resources that PDCLs (2) Lack of tumor microenvironment (3) Low take rates (4) Poor representation of well differentiated tumors	(1) Severely immunodeficient animals required (2) Stromal cells are rodent (3) Expensive and resource-intensive to scale (4) Low take rates (5) Poor representation of well-differentiated tumors
Recent advances and future direction	(1) Coculture systems with nontumor cells (2) Expanded number with LIMORE to over 81 PDCLs (3) Multiregional tumor sampling to capture intratumor heterogeneity	(1) Multicellular clusters rather than single cell suspension for increased take rates (2) Coculture systems with nontumor cells (3) Ex vivo culture systems of PCTS	(1) Rodent models with humanized immune system and humanized livers (2) Potential to combine locoregional and systemic therapies

Abbreviations: 3D, 3 dimensional; ECM, extracellular matrix; PDCLs, patient-derived cell lines; PCTS, precision cut tissue slices.

### Patient-Derived Cell Lines

PDCLs have been widely used in preclinical cancer biology research due to their utility in dissecting molecular mechanisms and testing drug sensitivity, as well as their relative ease of use and low cost. These lines can be used for high throughput screening to elucidate tumor-specific vulnerabilities to a rapidly escalating number of molecularly targeted cancer therapies. With increasing recognition of the enormous degree of genomic heterogeneity across human cancers, there has been considerable effort to generate large datasets that link the molecular and pharmacologic profiles of a large number of cell lines in order to identify biomarkers of response to therapies that would likely be missed when only testing a few tumor lines. 31 32 33 34 However, these efforts in liver cancer have been limited by several key factors. First and foremost has been the difficulty in culturing primary liver cancer cells, and, therefore, the inability to represent the full diversity of patient tumors. Until recently, there were approximately 30 HCC cell lines available and only a handful of CCA lines. The majority of these PDCLs were established in the 20 ^th^ century without molecular profiling available for the primary tumor tissue; therefore, it is not clear how well these cell lines recapitulated the source tumors. 32 In addition, several commonly used lines are not HCC (SKHEP, HepG2), and others are known to be contaminated with other human cell lines, particularly HeLa cells. 35 36

To address some of these concerns, Caruso et al. performed genomic, transcriptomic, and proteomic profiling, as well as drug screening, on 34 publicly available HCC PDCLs and compared the findings to profiling done on 821 primary HCC tumors. 37 Their results demonstrated that HCC PDCLs had similar molecular signatures to HCC patient tumors with a few important caveats. First, HCC PDCLs were derived from mostly Asian patients with viral hepatitis (55% hepatitis B virus [HBV], 33% hepatitis C virus). Second, the PDCLs were enriched for molecular signatures of the “proliferative” HCC signature, including more frequent mutations in TP53, AXIN1, and FGF19/CCND1. These existing HCC PDCLs underrepresent tumors with CTNNB1 mutations and “nonproliferative” transcriptional profile, most likely due to the bottleneck created by *in vitro* culture. Lastly, the PDCLs had a higher median mutation rate compared to primary tumors, suggesting selection for cells with greater genetic instability or the occurrence of genetic drift in culture. Qiu et al. sought to more directly evaluate HCC PDCL fidelity to parent tumors by developing nine novel HCC PDCLs with molecular comparison to the parent tumor through early passages of these primary cells and established PDCLs after >20 passages. 38 Whole-genome sequencing demonstrated that HCC PDCLs shared greater than 80% of genetic alterations with the matched primary HCCs, including single nucleotide variants (SNVs), copy number alterations (CNAs), and HBV integration sites. While additional mutations were seen in PDCLs (established > early passages) compared with the primary tumor, the cell line-specific mutations were unique to each cell line with no common mutations seen among the cell lines and no mutations found in any reported HCC driver gene. Instead, these mutations were enriched for extracellular matrix- and cell cycle-associated pathways likely reflecting the nature of *in vitro* culture. Expanding on this work, the Liver Cancer Model Repository (LIMORE) was established, combining publicly available and newly generated HCC PDCLs. 39 Using a ROCK inhibitor to facilitate attachment of primary cells and a TGFb inhibitor to inhibits mesenchymal cells, success rate of primary HCC culture increased to over 40% allowing for the development of an additional 50 HCC PDCLs, for a total of 81 cell lines included in the repository. This expansion increased the diversity of available HCC cell lines with 85% of HCC driver mutations covered by at least one PDCL and over 50% of HCC driver mutations covered by at least three PDCLs. While these cell lines still overrepresent Asian patients with HBV and the “proliferative” phenotype (Hoshida S1 and S2, TP53 and FGF19 mutations), around 30% had mutations in the Wnt/B-catenin pathway (CTNNB1, AXIN1, APC) with a “nonproliferative” transcriptional signature (Hoshida S3).

To address challenges with inter- and intra-tumoral heterogeneity, Gao et al. demonstrated that culturing patient-derived cells from multiregional sampling of HCC tumors enabled increased representation of the genetic and phenotypic heterogeneity of HCC and provides a platform to determine how this intra-tumoral heterogeneity impacts sensitivity due different therapies. 40 Comparing 55 HCC PDCLs from 10 tumors, the mean percentage of heterogeneous mutations was around 40% with fewer than half of driver alterations occurring early in HCC evolution. Importantly, of the druggable alterations found in two tumors, all were later branch events leading to differential sensitivity of subclones within one tumor, highlighting the complexity of targeted therapy in HCC. However, a drug screen of 28 compounds from their in-house library was able to identify drugs that were effective against all subclones, suggesting that this strategy may be a powerful tool for precision medicine approaches though more costly and labor intensive. Notably, the HCC tumors used in this study were all from resection specimens of patients with HBV-driven HCC—a limitation of HCC PDCLs in general as these approaches are limited by tissue availability. Resection specimens are inherently biased to solitary lesions, early-stage disease, and patients without advanced cirrhosis, leading to dearth of PDCLs that represent intermediate and advanced HCC patients—the patients most in of need targeted therapies. Even with an expanding number of HCC PDCLs, cell lines are inherently limited in their ability to retain tumor cell three-dimensional (3D) structure, cell-to-cell interactions, and the tumor microenvironment. Novel culture systems have been developed to minimize each of these limitations with a major technical advance coming with the development 3D culture systems.

### Patient-Derived Tumor Organoids

Over the past two decades, there has been an increased awareness of the importance of the 3D aspects of solid tumors in tumorigenesis, as well as in tumor cell proliferation, invasion, metastasis, and response to therapy. 41 42 43 44 45 46 47 While *in vivo* models are important tools for investigating these factors, they are limited by intrinsic differences between animal models and human biology, as well as increased cost and complexity of experimental design. Advances in 3D cell culture techniques, driven by a better understanding of extracellular matrix biology and regulation of stem cell niches/differentiation, have allowed for the development of PDTOs that preserve their histologic and molecular integrity over months in culture. 48 49 50 The 3D structure of the PDTOs enables modeling of *in vivo* conditions such as cell morphology, cell–cell interactions, and cell growth kinetics. Nutrient and oxygen conditions mimic cancer cell programs, including hypoxia and angiogenesis signaling activation, that are not seen in two-dimensional (2D) models. 46 51 Similarly, drug sensitivities may be dependent on the culture type, especially with cytostatic drugs, and drug sensitivities observed in PDTOs have better correlated with molecular alterations than drug sensitivities in PDCLs. 46 47 Importantly, PDTOs have demonstrated utility for high-throughput drug screening approaches that were previously only possible in 2D culture systems, although these approaches in PDTOs do require more technical expertise and resource investment. 52 53

#### Patient-Derived Liver Cancer Organoids

Huch et al. optimized conditions for the development of first mouse and then human liver organoids. 54 55 56 In brief, liver cells are isolated from liver tissue using collagenase–acutase digestion and then resuspended in a basement–membrane matrix (Matrigel or Basement Membrane Extract) as domes that are then submerged in culture media. The media is initially supplemented with a ROCK inhibitor to minimize apoptosis and promote proliferation, as well as Wnt and Noggin to prevent premature cell differentiation and promote the maintenance of stem/progenitor cells. Interestingly, these culture systems favor growth of primary human bile duct cells; however, these cells can be differentiated into functional hepatocytes. Building on this work, Broutier et al. optimized protocols that allowed for the establishment of primary liver cancer organoids from resection specimens and demonstrated that these models recapitulate the histologic architecture, genomic landscape, and transcriptomic and protein expression profiles of the parent tumor even after long-term expansion *in vitro* for up to 1 year. 57 Comparing whole-exome sequencing (WES) of the parent tumor tissue to early PDTOs (<2 months) and late PDTOs (>4 months) confirmed that tumoroid cultures retained >90 and >80%, respectively, of the genetic variants found in the parent tumor. Importantly, PDTOs from different patients showed differential drug sensitivities when treated with 29 different anticancer compounds. In addition, when PDTOs were implanted into immunocompromised mice, each recapitulated parent tissue histology, metastatic capability, and drug sensitivity. Notably, only eight tumoroid lines were generated during this initial study, limiting the generalizability of the results; however, it clearly established the broad range of translational potential for liver tumor organoids. Saito et al. further leveraged these techniques to establish additional organoid lines for 3/6 (50%) intrahepatic CCA and 1/5 (20%) gallbladder carcinomas. 58 Nuciforo et al. adapted the protocol to allow for development of PDTOs from liver cancer biopsies specimens with much more limited primary tissue, representing an important advance in the clinical utility of tumor organoids, as this allows inclusion of patients with unresectable tumors—the population most likely to receive systemic therapy and with the greatest unmet therapeutic needs. 59 In addition to confirming that PDTOs generated from patient biopsies recapitulate the histologic, genetic, and transcriptional profiles of the parent tissue, Nuciforo et al. also demonstrated that PDTOs are polyclonal and largely preserve the intratumor heterogeneity of patient biopsies. To further investigate the impact of tumor heterogeneity, Li et al. generated 27 PDTOs from 5 patients with primary liver cancer using multifocal tumor sampling of resection specimens. 60 The authors then tested drug response to 129 cancer drugs and highlighted the impact of inter- and intratumor heterogeneity on drug sensitivities by classifying drugs into four categories: (1) pan-effective; (2) intra-patient divergence; (3) inter-patient divergence; and (4) largely ineffective. Notably sorafenib and gemcitabine were two of the top interpatient divergent drugs and are clinically used in HCC and CCA, respectively, suggesting that better patient selection through functional testing could improve treatment outcomes. Overall, this approach would likely increase the chances of discovering clinically relevant phenotypes, though resection specimens or multifocal liver biopsies would be necessary. In addition, it remains unclear how much sampling per patient and/or tumor would be sufficient to capture clinically relevant heterogeneity.

#### Limitations of Patient-Derived Liver Cancer Organoids

Current liver cancer PDTOs remain limited in their utility for precision medicine approaches by their low derivation efficiency. Thus far, liver cancer PDTOs have been unable to overcome the low take rates also seen in PDCLs with only approximately 30% of primary tumors developing PDTOs regardless of tissue specimen source (resection or biopsy), noting that this rate is lower for HCC organoids than CCA organoids (26–50 vs. 50–75%, respectively). 57 58 59 61 62 63 64 Established HCC PDTOs are also enriched for poorly differentiated tumors with notably no HCC PDTOs generated from well differentiated (Edmondson Grade I) tumors using conventional protocols. 57 59 61 62 63 Dong et al. showed that using multicellular clusters that include tumor cells, stromal cells, and noncellular components increased take rates of HCC PDTOs to 65%, including one PDTO from a well differentiated tumor. 65 Maier et al. also found that using cell clusters improved establishment of CCA PDTOs. 66 While increasing expertise and standardization of tissue collection, preparation, and culture management have improved take rates, the heterogeneity in cancer stem cells, particularly in HCC, will likely require a deeper understanding of the drivers of tumor stem cell proliferation in order to represent the diversity of primary liver cancers. In addition, cell culture conditions still need to be optimize to allow for high throughput drug screening without impacting tumor cell biology. 52 53 65 67 Finally, while genetic and phenotypic drift in culture is less with PDTOs than traditional 2D cell lines, genetic and transcriptional discordance increases with time in culture and number of passages, and it is not yet known at what point these discrepancies become clinically relevant.

#### Future Directions of Patient-Derived Liver Cancer Organoids

Current HCC PDTOs have focused on expanding the epithelial component of tumors and are notably lacking in the cell populations and spatial organization that constitute the tumor microenvironment, limiting their clinical utility especially for the study of immune and antiangiogenic therapies, which are currently the standard of care for advanced disease. Several emerging coculture model systems have demonstrated promise in dissecting tumor–stromal cross talk. Coculture of HCC cell line-derived organoids with endothelial cells from human umbilical veins demonstrated endothelial cell differentiation and tubule network formation in the absence of exogenous growth factors (VEGF, SDF-1) with a hypoxic tumor core and true gradient penetration of drugs comparable to PDX tumors. 68 69 In addition, the endothelial-HCC interactions results in production of tumor necrosis factor signaling and proinflammatory cytokine production from HCC cells. Notably, vessel formation required a synthetic hydrogel specifically designed to have adhesion ligands and matrix metalloproteinase (MMP)-sensitive domains, and the vasculature network generally declined after 3 days of coculture due to absence of pericytes; however, this model was able to identify differences in the ability of antiangiogenic therapies to induce apoptosis of endothelial cells versus only prevent angiogenesis. 68 Zhou et al. demonstrated that coculture of HCC PDTOs with autologous tumor infiltrating lymphocytes (TILs) compared with autologous peripheral blood lymphocytes resulted in enhanced antitumor activity in a TIL coculture system, though only the TILs with the most cytolytic activity resulted in tumor regression *in vivo* . 61 Further studies are needed to dissect the clinical relevance of these *in vitro* findings in the *in vivo* setting. Similarly, cultures systems for CCA PDTOs have also focused on expanding the epithelial counterpart of the tumor despite knowledge that these tumors contain a high degree of stromal reaction and desmoplasia. 70 Interestingly, when CCA PDTOs were implanted into immunodeficient mice, the tumors displayed areas of desmoplastic stroma reaction, suggesting that tumor intrinsic features play a role in recapitulating this phenotype if the appropriate factors are present in the tumor microenvironment. 59 Notably, coculture with cancer-associated fibroblasts promoted liver cancer PDTO growth and conferred drug resistance via direct cell–cell contact and paracrine factors in both HCC and CCA PDTOs. 71

Even with these advances, PDTOs are inherently limited in their ability to represent the complex interactions of the *in vivo* tumor microenvironment and its impact on therapeutic response. Ex vivo culture of precision cut tissue slices (PCTS) can preserve the tissue structure, extracellular matrix composition, and cellular heterogeneity and function of liver tissue and has been used to study drug metabolism and fibrosis. However, these models are significantly limited by a functional life span of 24 to 48 hours that is thought to be due to cell death from hypoxia. 72 Innovative strategies to improve oxygenation through air–liquid interfaces or proprietary bioreactors have increased liver tissue viability to 7 to 8 days. 73 74 Jagatia et al. reported the first PCTS of human liver cancer and demonstrated that tumor morphology, stroma, TILs, and tumor immunophenotype could be maintained for at least 8 days *in vitro* . 75 Lastly, Collins et al. have shown that PCTS of cell line-derived HCC xenografts could be leveraged for scaled drug screening in the 96-well plate format. 76

### Patient-Derived Xenografts

While advances in 3D culture are improving our ability to mimic the tumor microenvironment *in vitro* , *in vivo* animal models remain the gold standard for investigation into tumor biology and response to therapy. Genetically engineered mouse models (GEMMs) have proven utility in studying of the impact of a single or combined genetic alteration(s) on tumorigenesis and tumor biology; however, these models lack the heterogeneity and complexity of the molecular alterations present in human HCC. 77 Chemotoxic models allow for autochthonous tumors that have greater genetic diversity than GEMMs with tumor vascularization that mirrors human tumors, but tumorigenesis is more variable with longer latency and by different mechanisms than those seen in patients. 78 79 These syngeneic models utilize immunocompetent animals and allow for investigations into interactions between HCC tumors and the immune system with a few notable limitations. First, there are known differences in immunotherapy targets for human and murine homologs, and several FDA-approved immunotherapies fail to bind to equivalent mouse targets. 80 Second, GEMM tumors often have limited immunogenicity and often require additional strategies to produce the immunosurveillance and immunotherapy responses seen in patients. 81 82 Lastly, there are notable recognized, and likely unrecognized, differences in both innate and adaptive immunity between the murine and human immune systems that can limit translatability of findings. 83

PDXs, in which human tumors are implanted into immunodeficient mice, recapitulate the heterogeneity of human tumors and patient responses to therapy and are frequently used in preclinical therapeutic trials. 84 85 Historically, liver cancer PDXs have largely been generated from patients with surgically cured, early-stage disease, likely representing less aggressive tumors that may never require the systemic therapies being investigated. 86 87 88 89 90 91 92 93 94 95 96 97 98 99 100 101 102 Recent studies demonstrate the potential to generate liver cancer PDXs from biopsy samples, allowing for the development of PDXs from patients with all stages of disease. 103 104 Historically, the lack of immune system in these models has limited their utility for informing pressing questions in immuno-oncology; however, advances in immunodeficient mice and our understanding of hematopoietic stem/progenitor cells has promoted the development of humanized mouse models that allow for the *in vivo* interaction of human immune cells and human tumors.

#### Patient-Derived Liver Cancer Xenografts in Immunocompromised Mice

**Fig. 2:**
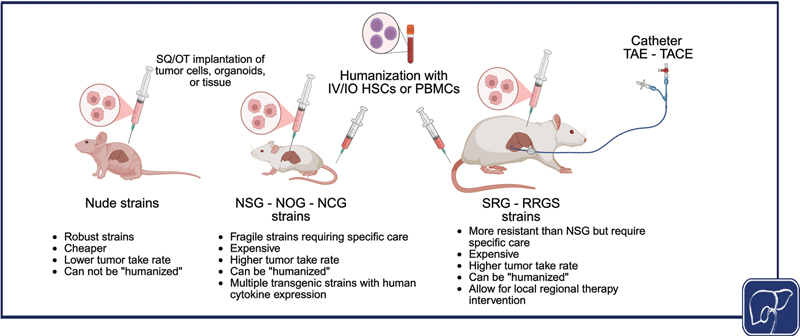
*In vivo* patient-derived models of liver cancer. Schematic of animal model systems used to implant liver cancer cell lines, organoids or tissue to model liver cancer *in vivo* . HSCs, hematopoietic stem cells; IV/IO, intravenous/intraosseous; PBMCs, peripheral blood mononuclear cells; SQ/OT, subcutaneous/orthotopic, TACE, transarterial chemoembolization; TAE, transarterial embolization. [rerif]. Source: *Created in BioRender. Simon, C. (2026)* *https://BioRender.com/uh0pvpu* .

The limitations of cell cultures systems, along with the increased availability of severely immunodeficient mice, have led to widespread use of PDXs for studying cancer biology and drug responsiveness. Patient-derived tumor cells or tissue can be implanted heterotopically in the flank using a subcutaneous injection or orthotopically in their organ of origin by surgical approaches ( Fig. 2 ). Xenograft models of primary liver cancer have included implantation of liver cancer cell lines, organoids, and patient cells or tissue. The first HCC PDX was established in 1996 via orthotopic implantation of HCC resection specimens in nude mice, noting the development of a PDX in only 1/30 (3.3%) mice. 86 Engraftment rates improved with the use of more immunodeficient mouse strains and tissue fragments rather than cell suspension; however, overall engraftment rates still remain low at around 20 to 40% in large cohorts. 62 91 93 98 103 The highest take rates have been seen in Nod- *scid* and Nod- *scid* IL2Rγ ^null^ (NSG) mice that lack functional B, T, and natural killer (NK) cells with impaired macrophages, dendritic cells, and complement response. 89 95 97 98 104 In addition, various mechanisms of liver injury, including partial hepatectomy or induction of cirrhosis, may improve PDX engraftment, underscoring the important role that the tumor microenvironment plays in regulating tumor cell proliferation. 91 100 102 105 Importantly, several studies have reported human lymphoma formation during the initial establishment of PDX models due to outgrowth of human lymphocytes infected with Epstein-Barr virus from patient liver cancer resection or biopsy specimens, highlighting the importance of histologic confirmation for all PDX lines. 89 103 104 Tischfield et al. demonstrated that implanting HCC tissue fragments in Matrigel reduces the risk of lymphoma formation from 2/4 PDXs (50%) to 1/7 PDXs (14%). 104 Xian et al. directly compared success rates of developing primary liver cancer PDTOs and PDXs from the same tissue samples and found no significant difference (29 vs. 24%, respectively), though notably this study was done using nude mice rather than NSG mice. 62 Success rates for CCA and cHCC–CCA were much higher for both PDTOs (9/17, 53% and 5/5, 100%) and PDXs (7/17, 41% and 3/5, 60%), likely reflecting the generally more aggressive tumor biology of these liver cancer subtypes.

Multiple studies have demonstrated the fidelity of liver cancer PDXs to their primary tumors in regard to histology, molecular profiles, clinical prognosis, and response to approved therapies. 62 93 94 96 98 100 103 104 Analysis of the histology of these tumors demonstrates that liver cancer PDXs retain the features of their parent tumor and maintain intertumor heterogeneity as evaluated by differentiation status, growth patterns, cytological subtypes, and liver cancer and hepatocyte/cholangiocyte markers by immunohistochemistry. 93 98 103 Liver cancer PDXs retain the genotypes of their parent tumor as measured by WES, short-tandem repeat analyses, and single-nucleotide polymorphism (SNP) arrays with a median of 85% (range: 65–100%) of somatic mutations in the HCC parent tumor expressed in the respective HCC PDX. 103 Notably, all missense mutations in HCC driver mutations were expressed in the corresponding PDX tumors stably over at least six PDX generations. With regard to gene expression profiles, HCC PDX tumors show downregulation of inflammatory and angiogenesis pathways, consistent with the loss of human immune cells and replacement of human vasculature and stroma with mouse vessels and stromal cells. 103 Importantly, the PDX tumors maintained the tumor-specific transcriptomic profiles of their parent tumors, and these profiles remained remarkably stable between the first and sixth generation of PDX tumors. These findings were consistent with prior data suggesting HCC PDXs have the least amount of genome discordance from their primary tumors compared to PDXs from multiple solid tumors in a large scale analysis of CNAs assessed by SNP arrays and WES. 106 Further supporting the fidelity of PDX to parent tumor, HCC PDXs have also been shown to predict patient prognosis, specifically the risk of relapse after resection and patient response to tyrosine kinase inhibitors. 100

#### Limitations of Patient-Derived Liver Cancer Xenografts in Immunocompromised Mice

A major limitation of PDX models is the limited scalability due the cost and resources required. In addition, the low derivation rate and underrepresentation of well-differentiated, “nonproliferative” subtype of HCC, similarly to liver cancer PDCLs and PDTOs, limits the generalizability of findings from liver cancer PDXs. 93 96 98 Interestingly, PDTOs are more enriched for aggressive cell types than PDXs based on expression of HCC stem cell markers in PDTOs and PDXs from the same parent tissue. 62 While PDXs better represent the interactions between tumor cells and the stromal and vasculature compartments, current models are still limited in their ability to recapitulate the phenotypes of chronic liver disease and cirrhosis seen in patients, as well as tumor–immune interactions given the lack of functional immune system. 78 Huang et al. demonstrated the impact of the tumor microenvironment on prognosis and therapeutic response by treating HCC PDXs in mice with and without cirrhosis. 100 Notably, metastases only developed in the cirrhotic mice and correlated with early recurrence postresection in patients. In addition, only the cirrhotic PDX model predicted patient responses to sorafenib and lenvatinib. Lastly, while PDXs have been leveraged to model ablation and external radiotherapy, HCC PDXs to date have been developed in mice, and their small size has prohibited investigation into endovascular LRTs. 107 108 109 Recent developments in immunodeficient rat strains have allowed for the implantation of PDX tumors into rats allowing for larger tumor development, more relevant pharmacokinetic and toxicology analysis, and the potential for investigation into endovascular LRTs. 110 111 112

#### Patient-Derived Liver Cancer Xenografts in Humanized Mice

Advances in immunodeficient rodents strains have resulted in not only more efficient PDX engraftment rates, but also the development of various humanized models that have become indispensable tools in modeling human-specific biological processes across a wide array of disciplines. 113 114 115 These humanized rodent models can be engrafted with human hematopoietic cells, human tissues, and human pathogens and have been leveraged for investigation into cancer pathogenesis, progression, immune evasion, and response to therapies, including ICIs, bispecific antibodies, and chimeric antigen receptor (CAR) T cells. 115 116 117 118 119 120 121 122 123 124 125 126

Most commonly “humanized mice” refers to immunodeficient mice xenotransplanted with human hematopoietic cells to generate mice with a humanized immune system (HIS). For immune–oncology applications, these HIS mice can then be implanted with PDX tumors to allow for human immune cell and human tumor cell interactions *in vivo* . There are several different HIS models mostly based on the source of human hematopoietic cells ( Fig. 2 ). Human peripheral blood mononuclear cells (PBMCs) or isolated T cells can be injected intravenously in NSG mice leading to good engraftment of memory and effector T cells; however, B cells and myeloid cells do not engraft well, and these mice uniformly develop xenogeneic graft-versus-host disease (xGVHD) within 2 months of implantation. 127 This model can be useful for evaluating therapies that suppress human T cell responses, such as antibody- or regulatory cell-based therapies or cytokines, in highly proliferative PDX tumors. Alternatively, engraftment with human hematopoietic stem and progenitor cells (HSPCs, i.e., CD34 ^+^ cells) from fetal livers, cord blood, or bone marrow result in HIS mice that produce all lineages of hematopoietic cells, including both innate and adaptive immune cells, with delayed onset of xGVHD to over 6 months posthumanization. 128 129 While lymphoid and myeloid progenitor cells engraft well in the bone marrow, there are low rates of mature innate immune cells in the periphery of these mice, including immune cell types important for tumor immune evasion such as macrophages, neutrophils, dendritic cells, and NK cells. 113 114 115 130 In addition, these models are limited by the lack of HLA expression for HLA-restricted T cell maturation and the relative absence of secondary lymphoid structures. 115 131 132 133

Next-generation humanized mice have been engineered to express various human cytokines and/or HLA proteins in order to overcome many of these limitations, including models that minimize the development of xGVHD and support the expansion and maturation of specific immune cell populations and lymphoid structures. 115 131 133 134 135 136 137 138 139 140 141 142 143 144 145 146 Each model has its own strengths and limitations that need to be considered in the context of the specific research question being asked. 115 Mouse strains genetically engineered to lack mouse major histocompatibility complex (MHC) class I and II molecules can be engrafted with PBMCs or isolated T cells with the onset of xGVHD delayed from 3 to 5 weeks to 8 to 11 weeks posthumanization without any deaths observed up to 14 weeks. 144 145 146 Building on this further, HUMAMICE were engineered to have deficient mouse MHC molecules and express human HLA molecules, reducing xGVHD and allowing for antigen-specific T and B cell responses when HLA-matched PBMCs were engrafted. 134 Alternatively, the coimplantation of human fetal liver HSPCs and fetal thymic tissue (BLT mice) also allows for T cell education on human rather than mouse MHC molecules. 137 147 148 BLT mice develop multilineage hematopoiesis, secondary lymphoid organs, and functional adaptive immune responses; however, this model is limited by the availability of human fetal liver and thymic tissue, limited development of the innate immune compartment, and earlier development of xGVHD than mice humanized with fetal liver cells alone. Similarly, two novel immunodeficient mice strains have been developed that restore the generation of lymphoid tissue inducer (LTi) cells and lymph node development through either the expression of IL-2R in LTi cells or expression of thymic stromal lymphopoietin in epithelial cells. 132 133 While these updated models improve lymphoid cell maturation and function, they do not address the limited development of the myeloid compartment in humanized models. To that end, immunodeficient mice were genetically engineered to express M-CSF, IL-3/GM-CSF, TPO ^+/−^ SIRPa (MITRG, MISTRG). 143 HIS MISTRG mice have high efficiency HSPC engraftment and support the development of functional and diverse innate immune cells, including monocytes, macrophages, and NK cells, with the caveat that increased phagocytosis of mouse erythroid cells leads to anemia in these models.

In the context of liver cancer, HCC PDXs have been used in several adopted cell transfer models using human PBMCs, T cells, and CAR T cells, though many investigators implant HCC cell lines rather than PDXs due to the rapid onset of xGVHD in these models. 121 122 123 Zhao et al. developed a HIS HCC PDX model that utilized human fetal liver-derived, partially HLA-matched HSPCs to humanize NSG mice and then HCC PDX tumors were implanted subcutaneously. 117 The model recapitulated key features of the HCC tumor immune microenvironment, specifically the upregulation of immune checkpoint markers on tumor cells, a T cell exhaustion phenotype in the TILs, and enrichment for tumor-associated macrophages (MØ2) and myeloid-derived suppressor cells. In addition, HCC PDX tumor growth was inhibited by ICIs in the HIS mice but not in the nonhumanized controls. Importantly, the humanized mice also exhibited immunotoxicity consistent with the immune-mediated adverse events that occur in patients. In a follow-up study, the HIS HCC PDX mouse model was successfully used to elucidate molecular and cellular pathways through which HCC tumor cells interacted with the immune system, as well as the effect of combination therapies. 118 Weinfurtner et al. demonstrated that utilizing an immunodeficient mouse strain that expresses human GM-CSF and IL-3 (NOG-EXL) increases overall tumor immune infiltration and the proportion of myeloid cells and regulatory immune cells, including MØ2 macrophages and regulatory T cells. 149 In addition, the model was further optimized for orthotopic rather than subcutaneous implantation of HCC PDX tumors and partial HLA-matching of HSPCs to the HCC PDX tumor using adult bone marrow-derived HSPCs. As this study and others demonstrated, using adult bone marrow-derived HSPCs requires more cells than HSPCs from fetal liver or cord blood, but these cells are more readily available in the quantities required for HLA-matching. 149 150 Due to supraphysiological levels of human GM-CSF and IL-3, all mice in this model eventually develop macrophage activation syndrome (MAS), a fatal condition similar to hemophagocytic lymphohistiocytosis seen in patients. 151 152 The onset and severity of MAS varies by mouse strain, degree of human chimerism, HSPC donor, and HCC PDX. 150 152 153 HIS MITRG/MISTRG mice do not develop MAS as the human cytokines are knocked-in to their respective mouse cytokine loci and are expressed at more physiologic levels; however, these strains are not commercially available and have not yet been implanted with HCC PDXs. 143

#### Ongoing Limitations and Future Directions of Humanized Patient-Derived Xenograft Models

HIS PDX models represent significant progress in the evolution of patient-derived models and the ability to study tumor and immune interactions *in vivo* ; however, there are several important limitations that currently hinder their scalability and broader application in translational research. The generation of these models is resource-intensive, requiring highly immunodeficient host strains, access to rare and ethically sensitive human tissues, and the use of specialized facilities with significant technical expertise. Given the logistical challenges of obtaining autologous bone marrow or mobilized peripheral blood from patients with cancer, most of these models are allografts rather than autografts with significant variability between donors in engraftment efficiency and immune reconstitution, which can reduce experimental consistency, throughput, and translatability. Importantly, there has been considerable effort to improve HSPC engraftment efficacy to reduce the amount of cells need and therefore cost of experiments, including new immunodeficient mouse strains, direct interosseus injections, and humanizing pups rather than adult mice. 153 154 155 Lastly, clinically relevant interventions such as locoregional therapies (e.g., endovascular LRTs) are not technically feasible in mice due to their small size and vascular anatomy. To overcome this limitation, there is growing interest in developing HIS models in larger animals, particularly in rats. 156

Overall, substantial progress has been made in the ability to model human tumor immunology in rodents, and the continued evolution of HIS PDX models will be critical for advancing translational therapeutics for cancer patients. Future efforts in HIS PDX models of primary liver cancer will focus on improved representation of the heterogeneity of patient tumors and the complexity of the human tumor microenvironment. Humanizing other elements of the tumor microenvironment, such as hepatocytes, hepatic stellate cells, and endothelial cells, has been shown to improve human immune cell reconstitution in the liver, including tissue resident immune cells (i.e. Kupffer cells). 157 158 In addition, HIS mice with humanized livers faithfully recapitulate features of chronic liver disease seen in patients, including alcohol-associated hepatitis, metabolic dysfunction-associated steatohepatitis, and viral hepatitis, allowing for a more accurate representation of the tumor microenvironment. 158 159 160 It is possible that mice with these liver microenvironments could improve engraftment rates for nonproliferative, well-differentiation subtypes of HCC that are underrepresented in current HCC PDXs. In addition, advances in gene editing using CRISPR/Cas9 could be leveraged to introduce specific driver mutations in human liver progenitor cells or PDX tumors prior to implantation in these humanized models to study the impact of specific mutations on tumor–immune cell interactions in the setting of varying causes of liver injury. Furthermore, the integration of HCC PDXs with autologous HSPCs could enable fully personalized humanized models for preclinical immunotherapy screening and assessment of individualized treatment regimens.

## Concluding Remarks

As the incidence of and mortality from primary liver cancer continue to rise globally, there is an urgent need for preclinical models that can faithfully recapitulate the molecular, immunologic, and microenvironment complexity of human disease. The development and refinement of patient-derived models, including cell lines, organoids, and xenografts, have significantly advanced our ability to study liver cancer in preclinical settings. As discussed in this review, each model offers unique advantages and limitations. More recently, the advancements in humanized rodent models provide an *in vivo* model to study tumor–immune interactions, assess immunotherapy responses, and investigate mechanisms of resistance; however, the full translational potential of these HIS models is currently limited by high cost, limited scalability, and incomplete immune reconstitution. Simultaneously, innovative coculture systems for PDTOs have dramatically improved our ability to reproduce the tumor microenvironment *in vitro* and have the potential to revolutionize high throughput drug screening. Together, these novel patient-derived model systems promise to bridge the gap between experimental findings and clinical phenotypes, offering a path forward toward more effective and personalized treatment strategies for primary liver cancer.

## Notes

Lay Summary Liver cancer is a growing global health issue with a high mortality rate. Although new treatments, like immunotherapy, have shown great promise in some patients, overall response rates remain low, and currently there are no biomarkers to guide treatment selection. To help address this problem and improve patient outcomes, better experimental models are needed. The present review describes various experimental models that use liver cancer tissue taken directly from patients. These patient-derived models can capture the diversity of liver cancer but only partially represent the complex environment in which these tumor cells grow. Advances in cell culture technique and animal model development have allowed for better modeling of how tumor cells interact with each other and the surrounding environment and may help improve our understanding of the disease. Ultimately, developing models from patient cancer tissue is essential for establishing more personalized and effective treatments for patient with liver cancer.

## References

[1] Bray F Laversanne M Sung H Global cancer statistics 2022: GLOBOCAN estimates of incidence and mortality worldwide for 36 cancers in 185 countries CA Cancer J Clin 2024 74 03 229 263 38572751 10.3322/caac.21834

[2] Rumgay H Arnold M Ferlay J Global burden of primary liver cancer in 2020 and predictions to 2040 J Hepatol 2022 77 06 1598 1606 36208844 10.1016/j.jhep.2022.08.021 PMC9670241

[3] Brar G Greten T F Graubard B I Hepatocellular carcinoma survival by etiology: a SEER-Medicare database analysis Hepatol Commun 2020 4 10 1541 1551 33024922 10.1002/hep4.1564 PMC7527688

[4] Jovel J Lin Z O'keefe S A survey of molecular heterogeneity in hepatocellular carcinoma Hepatol Commun 2018 2 08 941 955 30094405 10.1002/hep4.1197 PMC6078210

[5] Scudellari M Drug development: try and try again Nature 2014 516(7529):S4 S6 25470198 10.1038/516S4a

[6] Llovet J M Hernandez-Gea V Hepatocellular carcinoma: reasons for phase III failure and novel perspectives on trial design Clin Cancer Res 2014 20 08 2072 2079 24589894 10.1158/1078-0432.CCR-13-0547

[7] Llovet J M Villanueva A Lachenmayer A Finn R S Advances in targeted therapies for hepatocellular carcinoma in the genomic era Nat Rev Clin Oncol 2015 12 07 408 424 26054909 10.1038/nrclinonc.2015.103

[8] Mittal S El-Serag H B Sada Y H Hepatocellular carcinoma in the absence of cirrhosis in US Veterans is associated with non-alcoholic fatty liver disease Clin Gastroenterol Hepatol 2016 14 01 124 310 26196445 10.1016/j.cgh.2015.07.019 PMC4690789

[9] Villanueva A Llovet J M Liver cancer in 2013: mutational landscape of HCC–the end of the beginning Nat Rev Clin Oncol 2014 11 02 73 74 24395088 10.1038/nrclinonc.2013.243 PMC12261303

[10] Guichard C Amaddeo G Imbeaud S Integrated analysis of somatic mutations and focal copy-number changes identifies key genes and pathways in hepatocellular carcinoma Nat Genet 2012 44 06 694 698 22561517 10.1038/ng.2256 PMC3819251

[11] Schulze K Imbeaud S Letouzé E Exome sequencing of hepatocellular carcinomas identifies new mutational signatures and potential therapeutic targets Nat Genet 2015 47 05 505 511 25822088 10.1038/ng.3252 PMC4587544

[12] Yang C Zhang H Zhang L Evolving therapeutic landscape of advanced hepatocellular carcinoma Nat Rev Gastroenterol Hepatol 2023 20 04 203 222 36369487 10.1038/s41575-022-00704-9

[13] Llovet J M De Baere T Kulik L Locoregional therapies in the era of molecular and immune treatments for hepatocellular carcinoma Nat Rev Gastroenterol Hepatol 2021 18 05 293 313 33510460 10.1038/s41575-020-00395-0

[14] Llovet J M Bruix J Systematic review of randomized trials for unresectable hepatocellular carcinoma: chemoembolization improves survival Hepatology 2003 37 02 429 442 12540794 10.1053/jhep.2003.50047

[15] Salem R Gordon A C Mouli S Y90 radioembolization significantly prolongs time to progression compared with chemoembolization in patients with hepatocellular carcinoma Gastroenterology 2016 151 06 1155 116300 27575820 10.1053/j.gastro.2016.08.029 PMC5124387

[16] Brown A M Kassab I Massani M TACE versus TARE for patients with hepatocellular carcinoma: Overall and individual patient level meta analysis Cancer Med 2023 12 03 2590 2599 35943116 10.1002/cam4.5125 PMC9939158

[17] Xu M J Feng M Radiation therapy in HCC: what data exist and what data do we need to incorporate into guidelines? Semin Liver Dis 2019 39 01 43 52 30536291 10.1055/s-0038-1676098

[18] SHARP Investigators Study Group Llovet J M Ricci S Mazzaferro V Sorafenib in advanced hepatocellular carcinoma N Engl J Med 2008 359 04 378 390 18650514 10.1056/NEJMoa0708857

[19] Kudo M Finn R S Qin S Lenvatinib versus sorafenib in first-line treatment of patients with unresectable hepatocellular carcinoma: a randomised phase 3 non-inferiority trial Lancet 2018 391(10126):1163 1173 29433850 10.1016/S0140-6736(18)30207-1

[20] RESORCE Investigators Bruix J Qin S Merle P Regorafenib for patients with hepatocellular carcinoma who progressed on sorafenib treatment (RESORCE): a randomised, double-blind, placebo-controlled, phase 3 trial Lancet 2017 389(10064):56 66 27932229 10.1016/S0140-6736(16)32453-9

[21] Abou-Alfa G K Meyer T Cheng A L Cabozantinib in patients with advanced and progressing hepatocellular carcinoma N Engl J Med 2018 379 01 54 63 29972759 10.1056/NEJMoa1717002 PMC7523244

[22] IMbrave150 Investigators Finn R S Qin S Ikeda M Atezolizumab plus bevacizumab in unresectable hepatocellular carcinoma N Engl J Med 2020 382 20 1894 1905 32402160 10.1056/NEJMoa1915745

[23] HIMALAYA Investigators Abou-Alfa G K Lau G Kudo M Tremelimumab plus durvalumab in unresectable hepatoceullar carcinoma NEJM Evid 2022 1 08 a2100070 10.1056/EVIDoa2100070 38319892

[24] CheckMate 9DW investigators Yau T Galle P R Decaens T Nivolumab plus ipilimumab versus lenvatinib or sorafenib as first-line treatment for unresectable hepatocellular carcinoma (CheckMate 9DW): an open-label, randomised, phase 3 trial Lancet 2025 405(10492):1851 1864 40349714 10.1016/S0140-6736(25)00403-9

[25] Lin Z F Qin L X Chen J H Biomarkers for response to immunotherapy in hepatobiliary malignancies Hepatobiliary Pancreat Dis Int 2022 21 05 413 419 35973935 10.1016/j.hbpd.2022.08.002

[26] Roschke A V Tonon G Gehlhaus K S Karyotypic complexity of the NCI-60 drug-screening panel Cancer Res 2003 63 24 8634 8647 14695175

[27] Olivotto M Dello Sbarba P Environmental restrictions within tumor ecosystems select for a convergent, hypoxia-resistant phenotype of cancer stem cells Cell Cycle 2008 7 02 176 187 18256528 10.4161/cc.7.2.5315

[28] Daniel V C Marchionni L Hierman J S A primary xenograft model of small-cell lung cancer reveals irreversible changes in gene expression imposed by culture *in vitro* Cancer Res 2009 69 08 3364 3373 19351829 10.1158/0008-5472.CAN-08-4210 PMC2821899

[29] Gillet J P Calcagno A M Varma S Redefining the relevance of established cancer cell lines to the study of mechanisms of clinical anti-cancer drug resistance Proc Natl Acad Sci U S A 2011 108 46 18708 18713 22068913 10.1073/pnas.1111840108 PMC3219108

[30] Januszyk M Rennert R C Sorkin M Evaluation the effect of cell culture on gene expression in primary tissue samples using microfluidic-based single cell transcriptional analysis Microarrays (Basel) 2015 4 04 540 550 27600239 10.3390/microarrays4040540 PMC4996408

[31] Wilding J L Bodmer W F Cancer cell lines for drug discovery and development Cancer Res 2014 74 09 2377 2384 24717177 10.1158/0008-5472.CAN-13-2971

[32] Barretina J Caponigro G Stransky N The Cancer Cell Line Encyclopedia enables predictive modelling of anticancer drug sensitivity Nature 2012 483(7391):603 607 22460905 10.1038/nature11003 PMC3320027

[33] Garnett M J Edelman E J Heidorn S J Systematic identification of genomic markers of drug sensitivity in cancer cells Nature 2012 483(7391):570 575 22460902 10.1038/nature11005 PMC3349233

[34] Ghandi M Huang F W Jané-Valbuena J Next-generation characterization of the Cancer Cell Line Encyclopedia Nature 2019 569(7757):503 508 31068700 10.1038/s41586-019-1186-3 PMC6697103

[35] Rebouissou S Zucman-Rossi J Moreau R Qiu Z Hui L Note of caution: Contaminations of hepatocellular cell lines J Hepatol 2017 67 05 896 897 28807831 10.1016/j.jhep.2017.08.002

[36] Bian X Yang Z Feng H Sun H Liu Y A combination of species identification and STR profiling identifies cross-contaminated cells from 482 human tumor cell lines Sci Rep 2017 7 01 9774 28851942 10.1038/s41598-017-09660-w PMC5575032

[37] Caruso S Calatayud A L Pilet J Analysis of liver cancer cell lines identifies agents with likely efficacy against hepatocellular carcinoma and markers of response Gastroenterology 2019 157 03 760 776 31063779 10.1053/j.gastro.2019.05.001

[38] Qiu Z Zou K Zhuang L Hepatocellular carcinoma cell lines retain the genomic and transcriptomic landscapes of primary human cancers Sci Rep 2016 6 27411 27273737 10.1038/srep27411 PMC4895220

[39] Qiu Z Li H Zhang Z A pharmacogenomic landscape in human liver cancers Cancer Cell 2019 36 02 179 1.93E13 31378681 10.1016/j.ccell.2019.07.001 PMC7505724

[40] Gao Q Wang Z C Duan M Cell culture system for analysis of genetic heterogeneity within hepatocellular carcinomas and response to pharmacologic agents Gastroenterology 2017 152 01 232 2.42E6 27639803 10.1053/j.gastro.2016.09.008

[41] Bissell M J Radisky D Putting tumours in context Nat Rev Cancer 2001 1 01 46 54 11900251 10.1038/35094059 PMC2975572

[42] Bissell M J Kenny P A Radisky D C Microenvironmental regulators of tissue structure and function also regulate tumor induction and progression: the role of extracellular matrix and its degrading enzymes Cold Spring Harb Symp Quant Biol 2005 70 343 356 16869771 10.1101/sqb.2005.70.013 PMC3004779

[43] Aranda V Haire T Nolan M E Par6-aPKC uncouples ErbB2 induced disruption of polarized epithelial organization from proliferation control Nat Cell Biol 2006 8 11 1235 1245 17060907 10.1038/ncb1485

[44] Leslie K Gao S P Berishaj M Differential interleukin-6/Stat3 signaling as a function of cellular context mediates Ras-induced transformation Breast Cancer Res 2010 12 05 R80 20929542 10.1186/bcr2725 PMC3096973

[45] Lee G Y Kenny P A Lee E H Bissell M J Three-dimensional culture models of normal and malignant breast epithelial cells Nat Methods 2007 4 04 359 365 17396127 10.1038/nmeth1015 PMC2933182

[46] Imamura Y Mukohara T Shimono Y Comparison of 2D- and 3D-culture models as drug-testing platforms in breast cancer Oncol Rep 2015 33 04 1837 1843 25634491 10.3892/or.2015.3767

[47] Jabs J Zickgraf F M Park J Screening drug effects in patient-derived cancer cells links organoid responses to genome alterations Mol Syst Biol 2017 13 11 955 29180611 10.15252/msb.20177697 PMC5731348

[48] Sato T Vries R G Snippert H J Single Lgr5 stem cells build crypt-villus structures *in vitro* without a mesenchymal niche Nature 2009 459(7244):262 265 19329995 10.1038/nature07935

[49] Sato T Stange D E Ferrante M Long-term expansion of epithelial organoids from human colon, adenoma, adenocarcinoma, and Barrett's epithelium Gastroenterology 2011 141 05 1762 1772 21889923 10.1053/j.gastro.2011.07.050

[50] Rock J R Onaitis M W Rawlins E L Basal cells as stem cells of the mouse trachea and human airway epithelium Proc Natl Acad Sci U S A 2009 106 31 12771 12775 19625615 10.1073/pnas.0906850106 PMC2714281

[51] Däster S Amatruda N Calabrese D Induction of hypoxia and necrosis in multicellular tumor spheroids is associated with resistance to chemotherapy treatment Oncotarget 2017 8 01 1725 1736 27965457 10.18632/oncotarget.13857 PMC5352092

[52] Kondo J Ekawa T Endo H High-throughput screening in colorectal cancer tissue-originated spheroids Cancer Sci 2019 110 01 345 355 30343529 10.1111/cas.13843 PMC6317944

[53] Bergdorf K Phifer C Bharti V High-throughput drug screening of fine-needle aspiration-derived cancer organoids STAR Protoc 2020 1 03 100212 33377106 10.1016/j.xpro.2020.100212 PMC7757655

[54] Huch M Dorrell C Boj S F In vitro expansion of single Lgr5+ liver stem cells induced by Wnt-driven regeneration Nature 2013 494(7436):247 250 23354049 10.1038/nature11826 PMC3634804

[55] Huch M Gehart H van Boxtel R Long-term culture of genome-stable bipotent stem cells from adult human liver Cell 2015 160(1-2):299 312 25533785 10.1016/j.cell.2014.11.050 PMC4313365

[56] Broutier L Andersson-Rolf A Hindley C J Culture and establishment of self-renewing human and mouse adult liver and pancreas 3D organoids and their genetic manipulation Nat Protoc 2016 11 09 1724 1743 27560176 10.1038/nprot.2016.097

[57] Broutier L Mastrogiovanni G Verstegen M M Human primary liver cancer-derived organoid cultures for disease modeling and drug screening Nat Med 2017 23 12 1424 1435 29131160 10.1038/nm.4438 PMC5722201

[58] Saito Y Muramatsu T Kanai Y Establishment of patient-derived organoids and drug screening for biliary tract carcinoma Cell Rep 2019 27 04 1265 1.276E7 31018139 10.1016/j.celrep.2019.03.088

[59] Nuciforo S Fofana I Matter M S Organoid models of human liver cancers derived from tumor needle biopsies Cell Rep 2018 24 05 1363 1376 30067989 10.1016/j.celrep.2018.07.001 PMC6088153

[60] Li L Knutsdottir H Hui K Human primary liver cancer organoids reveal intratumor and interpatient drug response heterogeneity JCI Insight 2019 4 02 e121490 30674722 10.1172/jci.insight.121490 PMC6413833

[61] Zhou Z Yan X Shi W Evaluation of the tumoricidal efficacy of adoptive cell transfer using hepatocellular carcinoma-derived organoids J Gastrointest Oncol 2022 13 02 732 743 35557574 10.21037/jgo-21-715 PMC9086051

[62] Xian L Zhao P Chen X Heterogeneity, inherent and acquired drug resistance in patient-derived organoid models of primary liver cancer Cell Oncol (Dordr) 2022 45 05 1019 1036 36036881 10.1007/s13402-022-00707-3 PMC12978112

[63] Wang S Wang Y Xun X Hedgehog signaling promotes sorafenib resistance in hepatocellular carcinoma patient-derived organoids J Exp Clin Cancer Res 2020 39 01 22 31992334 10.1186/s13046-020-1523-2 PMC6986013

[64] Ren X Huang M Weng W Personalized drug screening in patient-derived organoids of biliary tract cancer and its clinical application Cell Rep Med 2023 4 11 101277 37944531 10.1016/j.xcrm.2023.101277 PMC10694672

[65] Dong H Li Z Bian S Culture of patient-derived multicellular clusters in suspended hydrogel capsules for pre-clinical personalized drug screening Bioact Mater 2022 18 164 177 35387168 10.1016/j.bioactmat.2022.03.020 PMC8961426

[66] Maier C F Zhu L Nanduri L K Patient-derived organoids of cholangiocarcinoma Int J Mol Sci 2021 22 16 8675 34445380 10.3390/ijms22168675 PMC8395494

[67] Ji S Feng L Fu Z Pharmaco-proteogenomic characterization of liver cancer organoids for precision oncology Sci Transl Med 2023 15 706 eadg3358 37494474 10.1126/scitranslmed.adg3358 PMC10949980

[68] Chiew G GY Wei N Sultania S Lim S Luo K Q Bioengineered three-dimensional co-culture of cancer cells and endothelial cells: a model system for dual analysis of tumor growth and angiogenesis Biotechnol Bioeng 2017 114 08 1865 1877 28369747 10.1002/bit.26297

[69] Lim J TC Kwang L G Ho N CW Hepatocellular carcinoma organoid co-cultures mimic angiocrine crosstalk to generate inflammatory tumor microenvironment Biomaterials 2022 284 121527 35483200 10.1016/j.biomaterials.2022.121527

[70] Sirica A E Gores G J Desmoplastic stroma and cholangiocarcinoma: clinical implications and therapeutic targeting Hepatology 2014 59 06 2397 2402 24123296 10.1002/hep.26762 PMC3975806

[71] Liu J Li P Wang L Cancer-associated fibroblasts provide a stromal niche for liver cancer organoids that confer trophic effects and therapy resistance Cell Mol Gastroenterol Hepatol 2021 11 02 407 431 32932015 10.1016/j.jcmgh.2020.09.003 PMC7788239

[72] de Graaf I AM Olinga P de Jager M H Preparation and incubation of precision-cut liver and intestinal slices for application in drug metabolism and toxicity studies Nat Protoc 2010 5 09 1540 1551 20725069 10.1038/nprot.2010.111

[73] Wu X Roberto J B Knupp A Precision-cut human liver slice cultures as an immunological platform J Immunol Methods 2018 455 71 79 29408707 10.1016/j.jim.2018.01.012 PMC6689534

[74] Paish H L Reed L H Brown H A bioreactor technology for modeling fibrosis in human and rodent precision-cut liver slices Hepatology 2019 70 04 1377 1391 30963615 10.1002/hep.30651 PMC6852483

[75] Jagatia R Doornebal E J Rastovic U Patient-derived precision cut tissue slices from primary liver cancer as a potential platform for preclinical drug testing EBioMedicine 2023 97 104826 37806285 10.1016/j.ebiom.2023.104826 PMC10667128

[76] Collins A L Kirkness K Ramon-Gil E Precision-cut tumor slices for modeling hepatocellular carcinoma enable at-scale drug screening Hepatol Commun 2025 9 06 e0706 40377490 10.1097/HC9.0000000000000706 PMC12088631

[77] Olson B Li Y Lin Y Liu E T Patnaik A Mouse models for cancer immunotherapy research Cancer Discov 2018 8 11 1358 1365 30309862 10.1158/2159-8290.CD-18-0044 PMC8725605

[78] Nevzorova Y A Boyer-Diaz Z Cubero F J Gracia-Sancho J Animal models for liver disease - a practical approach for translational research J Hepatol 2020 73 02 423 440 32330604 10.1016/j.jhep.2020.04.011

[79] Connor F Rayner T F Aitken S J Mutational landscape of a chemically-induced mouse model of liver cancer J Hepatol 2018 69 04 840 850 29958939 10.1016/j.jhep.2018.06.009 PMC6142872

[80] Magiera-Mularz K Kocik J Musielak B Human and mouse PD-L1: similar molecular structure, but different druggability profiles iScience 2020 24 01 101960 33437940 10.1016/j.isci.2020.101960 PMC7788105

[81] Ruiz de Galarreta M Bresnahan E Molina-Sánchez P B-catenin activation promotes immune escape and resistance to anti-PD-1 in hepatocellular carcinoma Cancer Discov 2019 9 08 1124 1141 31186238 10.1158/2159-8290.CD-19-0074 PMC6677618

[82] Wang J Perry C J Meeth K UV-induced somatic mutations elicit a functional T cell response in the YUMMER1.7 mouse melanoma model Pigment Cell Melanoma Res 2017 30 04 428 435 28379630 10.1111/pcmr.12591 PMC5820096

[83] Mestas J Hughes C CW Of mice and not men: differences between mouse and human immunology J Immunol 2004 172 05 2731 2738 14978070 10.4049/jimmunol.172.5.2731

[84] Gao H Korn J M Ferretti S High-throughput screening using patient-derived tumor xenografts to predict clinical trial drug response Nat Med 2015 21 11 1318 1325 26479923 10.1038/nm.3954

[85] Byrne A T Alférez D G Amant F Interrogating open issues in cancer precision medicine with patient-derived xenografts Nat Rev Cancer 2017 17 04 254 268 28104906 10.1038/nrc.2016.140

[86] Sun F X Tang Z Y Lui K D Establishment of a metastatic model of human hepatocellular carcinoma in nude mice via orthotopic implantation of histologically intact tissues Int J Cancer 1996 66 02 239 243 8603818 10.1002/(SICI)1097-0215(19960410)66:2<239::AID-IJC17>3.0.CO;2-7

[87] Armengol C Tarafa G Boix L Orthotopic implantation of human hepatocellular carcinoma in mice: analysis of tumor progression and establishment of the BCLC-9 cell line Clin Cancer Res 2004 10 06 2150 2157 15041736 10.1158/1078-0432.ccr-03-1028

[88] Huynh H Soo K C Chow P KH Panasci L Tran E Xenografts of human hepatocellular carcinoma: a useful model for testing drugs Clin Cancer Res 2006 12(14 Pt 1):4306 4314 16857806 10.1158/1078-0432.CCR-05-2568

[89] Chen K Ahmed S Adeyi O Dick J E Ghanekar A Human solid tumor xenografts in immunodeficient mice are vulnerable to lymphomagenesis associated with Epstein-Barr virus PLoS One 2012 7 06 e39294 22723990 10.1371/journal.pone.0039294 PMC3377749

[90] Yan M Li H Zhao F Establishment of NOD/SCID mouse models of human hepatocellular carcinoma via subcutaneous transplantation of histologically intact tumor tissue Chin J Cancer Res 2013 25 03 289 298 23825905 10.3978/j.issn.1000-9604.2013.05.02 PMC3696707

[91] Ahmed S U Zair M Chen K Generation of subcutaneous and intrahepatic human hepatocellular carcinoma xenografts in immunodeficient mice J Vis Exp 2013 25 79 e50544 10.3791/50544 PMC3935740 24121300

[92] Xin H Wang K Hu G Establishment and characterization of 7 novel hepatocellular carcinoma cell lines from patient-derived tumor xenografts PLoS One 2014 9 01 e85308 24416385 10.1371/journal.pone.0085308 PMC3887059

[93] Gu Q Zhang B Sun H Genomic characterization of a large panel of patient-derived hepatocellular carcinoma xenograft tumor models for preclinical development Oncotarget 2015 6 24 20160 20176 26062443 10.18632/oncotarget.3969 PMC4652995

[94] Zhao Q Zhou H Liu Q Prognostic value of the expression of cancer stem cell-related markers CD133 and CD44 in hepatocellular carcinoma: From patients to patient-derived tumor xenograft models Oncotarget 2016 7 30 47431 47443 27329727 10.18632/oncotarget.10164 PMC5216952

[95] Jiang Z Jiang X Chen S Anti-GPC3-CAR T cells suppress the growth of tumor cells in patient-derived xenografts of hepatocellular carcinoma Front Immunol 2017 7 690 28123387 10.3389/fimmu.2016.00690 PMC5225101

[96] He S Hu B Li C PDXliver: a database of liver cancer patient derived xenograft mouse models BMC Cancer 2018 18 01 550 29743053 10.1186/s12885-018-4459-6 PMC5944069

[97] Liu R Li Y Tian L Gankyrin drives metabolic reprogramming to promote tumorigenesis, metastasis and drug resistance through activating β-catenin/c-Myc signaling in human hepatocellular carcinoma Cancer Lett 2019 443 34 46 30503555 10.1016/j.canlet.2018.11.030

[98] Hu B Li H Guo W Establishment of a hepatocellular carcinoma patient-derived xenograft platform and its application in biomarker identification Int J Cancer 2020 146 06 1606 1617 31310010 10.1002/ijc.32564

[99] Nazzal M Sur S Steele R Establishment of a patient-derived xenograft tumor from hepatitis C-associated liver cancer and evaluation of imatinib treatment efficacy Hepatology 2020 72 02 379 388 32356575 10.1002/hep.31298 PMC7483967

[100] Huang D Q Muthiah M D Zhou L Predicting HCC response to multikinase inhibitors with *in vivo* cirrhotic mouse model for personalized therapy Cell Mol Gastroenterol Hepatol 2021 11 05 1313 1325 33340714 10.1016/j.jcmgh.2020.12.009 PMC8020437

[101] Wu Y Wang J Zheng X Establishment and preclinical therapy of patient-derived hepatocellular carcinoma xenograft model Immunol Lett 2020 223 33 43 32335145 10.1016/j.imlet.2020.04.009

[102] Zhu M Li L Lu T Uncovering biological factors that regulate hepatocellular carcinoma growth using patient-derived xenograft assays Hepatology 2020 72 03 1085 1101 31899548 10.1002/hep.31096 PMC7332388

[103] Blumer T Fofana I Matter M S Hepatocellular carcinoma xenografts established from needle biopsies preserve the characteristics of the originating tumors Hepatol Commun 2019 3 07 971 986 31334445 10.1002/hep4.1365 PMC6601318

[104] Tischfield D J Ackerman D Noji M Establishment of hepatocellular carcinoma patient-derived xenografts from image-guided percutaneous biopsies Sci Rep 2019 9 01 10546 31332214 10.1038/s41598-019-47104-9 PMC6646301

[105] Zou C El Dika I Vercauteren K OA Mouse characteristics that affect establishing xenografts from hepatocellular carcinoma patient biopsies in the United States Cancer Med 2022 11 03 602 617 34951132 10.1002/cam4.4375 PMC8817074

[106] Hoge A CH Getz M Zimmer A DNA-based copy number analysis confirms genomic evolution of PDX models NPJ Precis Oncol 2022 6 01 30 35484194 10.1038/s41698-022-00268-6 PMC9050710

[107] Su T Liao J Dai Z Stress-induced phosphoprotein 1 mediates hepatocellular carcinoma metastasis after insufficient radiofrequency ablation Oncogene 2018 37 26 3514 3527 29559743 10.1038/s41388-018-0169-4

[108] Yeoh K W Prawira A Saad M ZB Vinorelbine augments radiotherapy in hepatocellular carcinoma Cancers (Basel) 2020 12 04 872 32260169 10.3390/cancers12040872 PMC7226273

[109] Zhou K Jiang Y Feng S Establishment of image-guided radiotherapy of orthotopic hepatocellular carcinoma mouse model Animal Model Exp Med 2023 6 05 419 426 37365733 10.1002/ame2.12335 PMC10614124

[110] He D Zhang J Wu W A novel immunodeficient rat model supports human lung cancer xenografts FASEB J 2019 33 01 140 150 29944447 10.1096/fj.201800102RR

[111] Noto F K Sangodkar J Adedeji B T The SRG rat, a Sprague-Dawley Rag2/Il2rg double-knockout validated for human tumor oncology studies PLoS One 2020 15 10 e0240169 33027304 10.1371/journal.pone.0240169 PMC7540894

[112] Gade T PF Hunt S J Harrison N Segmental TAE in a translational rat model of hepatocellular carcinoma J Vasc Interv Radiol 2015 26 08 1 9 25863596 10.1016/j.jvir.2015.02.006 PMC4853022

[113] Shultz L D Brehm M A Garcia-Martinez J V Greiner D L Humanized mice for immune system investigation: progress, promise and challenges Nat Rev Immunol 2012 12 11 786 798 23059428 10.1038/nri3311 PMC3749872

[114] Walsh N C Kenney L L Jangalwe S Humanized mouse models of clinical disease Annu Rev Pathol 2017 12 187 215 27959627 10.1146/annurev-pathol-052016-100332 PMC5280554

[115] Chuprin J Buettner H Seedhom M O Humanized mouse models for immuno-oncology research Nat Rev Clin Oncol 2023 20 03 192 206 36635480 10.1038/s41571-022-00721-2 PMC10593256

[116] Marín-Jiménez J A Capasso A Lewis M S Testing cancer immunotherapy in a human immune system mouse model: correlating treatment responses to human chimerism, therapeutic variables, and immune cell phenotypes Front Immunol 2021 12 607282 33854497 10.3389/fimmu.2021.607282 PMC8040953

[117] Zhao Y Shuen T WH Toh T B Development of a new patient-derived xenograft humanised mouse model to study human-specific tumour microenvironment and immunotherapy Gut 2018 67 10 1845 1854 29602780 10.1136/gutjnl-2017-315201 PMC6145285

[118] Zhao Y Wang J Liu W N Analysis and validation of human targets and treatments using a hepatocellular carcinoma immune humanized mouse model Hepatology 2021 74 03 1395 1410 33738839 10.1002/hep.31812 PMC9540409

[119] Horowitz N B Mohammad I Moreno-Nieves U Y Koliesnik I Tran Q Sunwoo J B Humanized mouse models for the advancement of innate lymphoid cell-based cancer immunotherapies Front Immunol 2021 12 648580 33968039 10.3389/fimmu.2021.648580 PMC8100438

[120] Mhaidly R Verhoeyen E Humanized mice are precious tools for preclinical evaluation of CAR T and CAR NK cell therapies Cancers (Basel) 2020 12 07 1915 32679920 10.3390/cancers12071915 PMC7409195

[121] Yu L Yang X Huang N A novel targeted GPC3/CD3 bispecific antibody for the treatment hepatocellular carcinoma Cancer Biol Ther 2020 21 07 597 603 32240054 10.1080/15384047.2020.1743158 PMC7515540

[122] Du K Li Y Liu J A bispecific antibody targeting GPC3 and CD47 induced enhanced antitumor efficacy against dual antigen-expressing HCC Mol Ther 2021 29 04 1572 1584 33429083 10.1016/j.ymthe.2021.01.006 PMC8058486

[123] Habibollahi P Gurevich A Hui J Z Integrated imaging probe and bispecific antibody development enables *in vivo* targeting of glypican-3-expressing hepatocellular carcinoma Mol Cancer Ther 2024 23 12 1815 1826 39312187 10.1158/1535-7163.MCT-23-0470 PMC11726262

[124] Barrett D M Zhao Y Liu X Treatment of advanced leukemia in mice with mRNA engineered T cells Hum Gene Ther 2011 22 12 1575 1586 21838572 10.1089/hum.2011.070 PMC3237694

[125] Li D Li N Zhang Y F Persistent polyfunctional chimeric antigen receptor T cells that target glypican 3 eliminate orthotopic hepatocellular carcinomas Gastroenterology 2020 158 08 2250 2.265E23 32060001 10.1053/j.gastro.2020.02.011 PMC7282931

[126] Lu L L Xiao S X Lin Z Y GPC3-IL7-CCL19-CAR-T primes immune microenvironment reconstitution for hepatocellular carcinoma therapy Cell Biol Toxicol 2023 39 06 3101 3119 37853185 10.1007/s10565-023-09821-w

[127] King M A Covassin L Brehm M A Human peripheral blood leucocyte non-obese diabetic-severe combined immunodeficiency interleukin-2 receptor gamma chain gene mouse model of xenogeneic graft-versus-host-like disease and the role of host major histocompatibility complex Clin Exp Immunol 2009 157 01 104 118 19659776 10.1111/j.1365-2249.2009.03933.x PMC2710598

[128] Shultz L D Lyons B L Burzenski L M Human lymphoid and myeloid cell development in NOD/LtSz-scid IL2R gamma null mice engrafted with mobilized human hemopoietic stem cells J Immunol 2005 174 10 6477 6489 15879151 10.4049/jimmunol.174.10.6477

[129] Sonntag K Eckert F Welker C Chronic graft-versus-host-disease in CD34(+)-humanized NSG mice is associated with human susceptibility HLA haplotypes for autoimmune disease J Autoimmun 2015 62 55 66 26143958 10.1016/j.jaut.2015.06.006

[130] Tanaka S Saito Y Kunisawa J Development of mature and functional human myeloid subsets in hematopoietic stem cell-engrafted NOD/SCID/IL2rγKO mice J Immunol 2012 188 12 6145 6155 22611244 10.4049/jimmunol.1103660 PMC3370073

[131] Danner R Chaudhari S N Rosenberger J Expression of HLA class II molecules in humanized NOD.Rag1KO.IL2RgcKO mice is critical for development and function of human T and B cells PLoS One 2011 6 05 e19826 21611197 10.1371/journal.pone.0019826 PMC3096643

[132] Halkias J Yen B Taylor K T Conserved and divergent aspects of human T-cell development and migration in humanized mice Immunol Cell Biol 2015 93 08 716 726 25744551 10.1038/icb.2015.38 PMC4575952

[133] Li Y Masse-Ranson G Garcia Z A human immune system mouse model with robust lymph node development Nat Methods 2018 15 08 623 630 30065364 10.1038/s41592-018-0071-6

[134] Zeng Y Liu B Rubio M T Creation of an immunodeficient HLA-transgenic mouse (HUMAMICE) and functional validation of human immunity after transfer of HLA-matched human cells PLoS One 2017 12 04 e0173754 28399128 10.1371/journal.pone.0173754 PMC5388326

[135] Brehm M A Kenney L L Wiles M V Lack of acute xenogeneic graft- versus-host disease, but retention of T-cell function following engraftment of human peripheral blood mononuclear cells in NSG mice deficient in MHC class I and II expression FASEB J 2019 33 03 3137 3151 30383447 10.1096/fj.201800636R PMC6404556

[136] Shultz L D Saito Y Najima Y Generation of functional human T-cell subsets with HLA-restricted immune responses in HLA class I expressing NOD/SCID/IL2r gamma(null) humanized mice Proc Natl Acad Sci U S A 2010 107 29 13022 13027 20615947 10.1073/pnas.1000475107 PMC2919921

[137] Lan P Tonomura N Shimizu A Wang S Yang Y G Reconstitution of a functional human immune system in immunodeficient mice through combined human fetal thymus/liver and CD34+ cell transplantation Blood 2006 108 02 487 492 16410443 10.1182/blood-2005-11-4388

[138] Li Y Teteloshvili N Tan S Humanized mice reveal new insights into the thymic selection of human autoreactive CD8+ T cells Front Immunol 2019 10 63 30778347 10.3389/fimmu.2019.00063 PMC6369192

[139] Takahashi T Katano I Ito R Enhanced antibody response in a novel NOG transgenic mouse with restored lymph node organogenesis Front Immunol 2018 8 2017 29387068 10.3389/fimmu.2017.02017 PMC5776085

[140] Billerbeck E Barry W T Mu K Development of human CD4+FoxP3+ regulatory T cells in SGM3 humanized mice Blood 2011 117 11 3076 3086 21252091 10.1182/blood-2010-08-301507 PMC3062310

[141] Maser I P Hoves S Bayer C The tumor milieu promotes functional human tumor-resident plasmacytoid dendritic cells in humanized mouse models Front Immunol 2020 11 2082 33013879 10.3389/fimmu.2020.02082 PMC7507800

[142] Herndler-Brandstetter D Shan L Yao Y Humanized mouse model supports development, function, and tissue residency of human natural killer cells Proc Natl Acad Sci U S A 2017 114 45 E9626 E9634 29078283 10.1073/pnas.1705301114 PMC5692533

[143] Rongvaux A Willinger T Martinek J Development and function of human innate immune cells in a humanized mouse model Nat Biotechnol 2014 32 04 364 372 24633240 10.1038/nbt.2858 PMC4017589

[144] Yaguchi T Kobayashi A Inozume T Human PBMC-transferred murine MHC class I/II-deficient NOG mice enable long-term evaluation of human immune responses Cell Mol Immunol 2018 15 11 953 962 29151581 10.1038/cmi.2017.106 PMC6207709

[145] Ashizawa T Iizuka A Nonomura C Antitumor effect of PD-1 blockade in humanized the NOG-MHC double knockout mouse Clin Cancer Res 2017 23 01 149 158 27458246 10.1158/1078-0432.CCR-16-0122

[146] Ka Y Katano I Nishinaka E Improved engraftment of human PBMCs in NOG MHC double knockout mice generated using CRISPR/Cas9 Immunol Letters 2021 229 55 61 10.1016/j.imlet.2020.11.011 33253759

[147] Lockridge J L Zhou Y Becker Y A Mice engrafted with human fetal thymic tissue and hematopoietic stem cells develop pathology resembling chronic graft-versus-host disease Biol Blood Marrow Transplant 2013 19 09 1310 1322 23806772 10.1016/j.bbmt.2013.06.007 PMC3755109

[148] Madley R Nauman G Danzl N Negative selection of human T cells recognizing a naturally-expressed tissue-restricted antigen in the human thymus J Transl Autoimmun 2020 3 100061 32875283 10.1016/j.jtauto.2020.100061 PMC7451786

[149] Weinfurtner K Tischfield D McClung G Human GM-CSF/IL-3 enhance tumor immune infiltration in humanized HCC patient-derived xenografts JHEP Rep Innov Hepatol 2024 7 03 101264 10.1016/j.jhepr.2024.101264 PMC11869099 40028346

[150] Lepus C M Gibson T F Gerber S A Comparison of human fetal liver umbilical cord blood, and adult blood hematopoietic stem cell engraftment in NSG, BRG, and C.B.-17- *scid* immunodeficient mice Hum Immunol 2009 70 10 790 802 19524633 10.1016/j.humimm.2009.06.005 PMC2949440

[151] Tarrant J C Binder Z A Bugatti M Pathology of macrophage activation syndrome in humanized NSGS mice Res Vet Sci 2021 134 137 146 33383491 10.1016/j.rvsc.2020.12.003

[152] Willis E Verrelle J Banerjee E Humanization with CD34-positive hematopoietic stem cells in NOG-EXL mice results in improved long-term survival and less severe myeloid cell hyperactivation phenotype relative to NSG-SGM3 mice Vet Pathol 2024 61 04 664 674 38197423 10.1177/03009858231222216 PMC11264550

[153] McIntosh B E Brown M E Duffin B M Nonirradiated NOD,B6.SCID Il2rγ-/- Kit(W41/W41) (NBSGW) mice support multilineage engraftment of human hematopoietic cells Stem Cell Reports 2015 4 02 171 180 25601207 10.1016/j.stemcr.2014.12.005 PMC4325197

[154] Yahata T Ando K Sato T A highly sensitive strategy for SCID-repopulating cell assay by direct injection of primitive human hematopoietic cells into NOD/SCID mice bone marrow Blood 2003 101 08 2905 2913 12411299 10.1182/blood-2002-07-1995

[155] Brehm M A Cuthbert A Yang C Parameters for establishing humanized mouse models to study human immunity: analysis of human hematopoietic stem cell engraftment in three immunodeficient strains of mice bearing the IL2rgamma(null) mutation Clin Immunol 2010 135 01 84 98 20096637 10.1016/j.clim.2009.12.008 PMC2835837

[156] Ménoret S Ouisse L H Tesson L *In vivo* analysis of human immune response in immunodeficient rats Transplantation 2020 104 04 715 723 31764762 10.1097/TP.0000000000003047 PMC7147402

[157] Guo J Li Y Shan Y Humanized mice reveal an essential role for human hepatocytes in the development of the liver immune system Cell Death Dis 2018 9 06 667 29867111 10.1038/s41419-018-0720-9 PMC5986801

[158] AlcHepNet Kaffe E Roulis M Zhao J Humanized mouse liver reveals endothelial control of essential hepatic metabolic functions Cell 2023 186 18 3793 3.809E29 37562401 10.1016/j.cell.2023.07.017 PMC10544749

[159] Kaffe E Mehal W ZGeneration and characterization of a humanized mouse model of alcohol-induced steatosis, inflammation, and fibrosis. AASLD Liver Meeting 2023. Abstract 46

[160] Keng C T Sze C W Zheng D Characterisation of liver pathogenesis, human immune responses and drug testing in a humanised mouse model of HCV infection Gut 2016 65 10 1744 1753 26149491 10.1136/gutjnl-2014-307856 PMC5036242

